# Bivartect: accurate and memory-saving breakpoint detection by direct read comparison

**DOI:** 10.1093/bioinformatics/btaa059

**Published:** 2020-01-27

**Authors:** Keisuke Shimmura, Yuki Kato, Yukio Kawahara

**Affiliations:** Department of RNA Biology and Neuroscience, Graduate School of Medicine, Osaka University, Suita, Osaka 565-0871, Japan

## Abstract

**Motivation:**

Genetic variant calling with high-throughput sequencing data has been recognized as a useful tool for better understanding of disease mechanism and detection of potential off-target sites in genome editing. Since most of the variant calling algorithms rely on initial mapping onto a reference genome and tend to predict many variant candidates, variant calling remains challenging in terms of predicting variants with low false positives.

**Results:**

Here we present Bivartect, a simple yet versatile variant caller based on direct comparison of short sequence reads between normal and mutated samples. Bivartect can detect not only single nucleotide variants but also insertions/deletions, inversions and their complexes. Bivartect achieves high predictive performance with an elaborate memory-saving mechanism, which allows Bivartect to run on a computer with a single node for analyzing small omics data. Tests with simulated benchmark and real genome-editing data indicate that Bivartect was comparable to state-of-the-art variant callers in positive predictive value for detection of single nucleotide variants, even though it yielded a substantially small number of candidates. These results suggest that Bivartect, a reference-free approach, will contribute to the identification of germline mutations as well as off-target sites introduced during genome editing with high accuracy.

**Availability and implementation:**

Bivartect is implemented in C^++^ and available along with *in silico* simulated data at https://github.com/ykat0/bivartect.

**Supplementary information:**

Supplementary data are available at *Bioinformatics* online.

## 1 Introduction

Genomic structural variations have been widely investigated at base pair resolution using the prevailing high-throughput sequencing technologies (Alkan *et al.*, 2011). Examples where genomic variations occur include germline/somatic mutations, ranging from single nucleotide variants (SNVs) to structural variants (SVs) of at least 50 bp (Sudmant *et al.*, 2015; The 1000 Genomes Project Consortium, 2015) such as insertions/deletions (indels), inversions or translocations (Weischenfeldt *et al.*, 2013). Given that these variants can be associated with complex diseases and somatic mutations may correlate with the progression of cancer, detection of these variants is essential to elucidate disease mechanisms (Martincorena and Campbell, 2015). In addition, the demand for detecting potential off-target sites with high accuracy is increasing as the use of genome-editing technologies increases (Kuscu *et al.*, 2014).

Most of the *in silico* methods for variant detection rely on initial mapping onto a reference genome (Chen *et al.*, 2009, 2016; Cibulskis *et al.*, 2013; DePristo *et al.*, 2011; Kim *et al.*, 2018; Lai *et al.*, 2016; Larson *et al.*, 2012; Rausch *et al.*, 2012; Wang *et al.*, 2011; Ye *et al.*, 2009). This implies that the quality of variant calling could be exacerbated when variant-containing reads are to be of no consideration due to being unmapped onto the reference genome. Additionally, these approaches typically predict many variant candidates and require some complex filtering steps based on statistical methods after the initial mapping in order to remove false-positive predictions, which would in turn result in lower sensitivity.

In contrast, some mapping-free approaches have been developed (Audano *et al.*, 2018; Iqbal *et al.*, 2012; Pajuste *et al.*, 2017; Rahman *et al.*, 2018; Standage *et al.*, 2019) on the basis of short substrings of length *k* called *k*-mers. These methods, however, require reference *k*-mers, abundant unique read *k*-mers, or known variant *k*-mers to detect novel variants. A method for direct comparison of sequence reads without focusing on specific *k*-mers has also been proposed (Moncunill *et al.*, 2014), which employs a quaternary sequence tree to expand all sequence reads together with their suffixes to be compared. This results in a huge memory requirement, and makes it impossible to run on a computer with a single node for analyzing omics data.

**Fig. 1.**
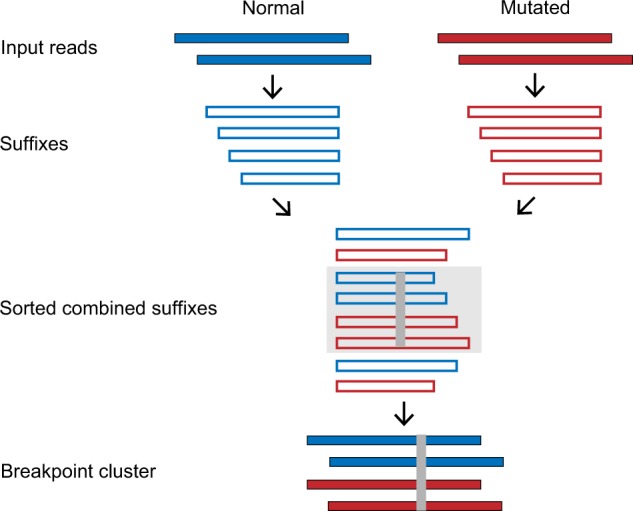
Overview of Bivartect for detecting potential breakpoints. This schema is depicted using single-end reads for simplicity. As the first step, suffixes of at least predefined length are derived from input normal/mutated reads. They are then combined and lexicographically sorted. The light shaded suffixes have a common prefix on the left of the dark gray column, which corresponds to a potential breakpoint. Finally, a breakpoint cluster is constructed by recovering reads from the corresponding suffixes to infer its variant type. (Color version of this figure is available at *Bioinformatics* online.)

To circumvent these problems, we developed Bivartect (bit-based variant detection), a simple yet accurate computational approach to detecting genomic variants based on direct comparison of sequence reads, which skips initial mapping (Fig. 1). To reduce memory use, Bivartect converts all sequences into bit strings, and keeps only a small part of the suffixes of the reads in the memory space during identification of breakpoints, which are defined as positions at which aligned sequences begin to differ. Moreover, to attain a speed-up and save memory use further to run on a computer with a single node, we adopted a strategy where part of the suffixes of the normal and mutated reads with a common prefix are sorted to detect potential breakpoints. This is repeated until all combinations of the prefixes are covered. Reads recovered from suffixes in a breakpoint cluster are then assembled to generate consensus sequences in both normal and mutated sequence groups, which are then used to infer a variant type with respect to the breakpoint (Fig. 1). If necessary, consensus normal sequences in distinct breakpoint clusters, whose mutated counterparts are predicted to have variants, are mapped onto a reference genome to identify the genomic location of those variants. Since Bivartect basically aims to find breakpoints along with consensus normal/mutated sequences as described earlier, it can detect not only SNVs but also indels, inversions and their complexes. In particular, Bivartect can detect SNVs and small indels with high accuracy, part of which was demonstrated in our computational test.

## 2 Materials and methods

### 2.1 The Bivartect pipeline

Assume that reads from normal/mutated samples, called normal/mutated reads for short, are given, and barcodes embedded in the reads are removed if necessary. Note that filtering a base by its quality is disallowed in our framework. The key steps of the workflow of Bivartect are illustrated in Figure 1. Briefly, the process of variant detection consists of the following steps: (i) when normal/mutated reads are input to Bivartect, they are converted into bit strings, all of which are stored in memory, and then divided into $4^{k}$ (e.g. *k* =* *3) small sets of their suffixes with a common prefix of length *k* to be processed sequentially. (ii) Bivartect seeks to sort suffixes in one of the above small sets, comprising both normal and mutated sequences, to construct a read cluster that contains a potential breakpoint. (iii) Consensus sequences for normal and mutated representatives are computed and compared with each other to infer the variant type. (iv) Steps (ii) and (iii) are iterated $4^{k}$ times to cover all possible common prefixes.

### 2.2 Storing read information in memory

To select a necessary subset of reads for computation as fast as possible and make their size as small as possible, all sequence reads are converted into bit strings where each base is represented by 2 bits (cf. 8 bits per base as a usual case). For example, ‘A,’ ‘C,’ ‘G’ and ‘T’ are converted into ‘00,’ ‘01,’ ‘10’ and ‘11,’ respectively. In addition, a unique ID is assigned to each read to enable short-duration access to the read. More specifically, 64 reads are grouped together to make a block, and header information for each read represented by 2 bytes is added to the head of the block, which stores an offset of the read concerned within the block. It is to be noted that naïve storing of an address of a read in memory requires 8 bytes in general. In contrast, in our implementation, only an addition of size 2+(8/64)=2.125 bytes per read is sufficient for access to the read, achieving fast access speed while reducing memory use.

### 2.3 Building a breakpoint cluster by divide-and-conquer

This part is divided into $4^{k}$ sub-processes, each processed sequentially to save memory, and merged into the final results. The partition is based on the pattern of a common prefix of length *k* in read suffixes (a suffix of a sequence is defined as a substring of the sequence that ends with the last letter of the sequence). For an example of a case where *k* =* *3 (i.e. 64 partitions) and the lexicographical order (in the lexicographical order, sequences are ordered according to the alphabetical order of their components; e.g. A, C, G and T from head to tail) is taken into account, suffixes beginning with ‘AAA’ are processed, and then the memory space necessary for this computation is released. Next, suffixes beginning with ‘AAC’ are processed, followed by releasing the memory that had been necessary for this process. These steps are repeated until the prefix ‘TTT’ is considered. It should be noted that the value of *k* does not affect the final results, and we used *k* = * *3 throughout the computational tests presented in this study.

Each sub-process is based on the following steps: (i) substrings beginning with a common prefix of length *k* are searched through all reads in memory, and suffixes with the prefix in the substrings are generated and stored in memory as additional components for subsequent analysis. (ii) Suffixes from normal reads are combined with suffixes from mutated reads, and lexicographically sorted. It is to be noted that information on whether each suffix is from a normal or mutated read is properly kept after the merger. (iii) Suffixes with the same prefix of length *d* are clustered when looking them up in the sorted list of suffixes from head to tail. Note that *d* is predefined as the shortest length of suffixes. (iv) A cluster with different bases starting at position *d* +* *1 is registered as a potential breakpoint cluster. (v) Reads are recovered from the corresponding suffixes in a breakpoint cluster. (vi) All suffixes with the common prefix of length *k* in memory are released. If the input is a set of non-strand-specific paired-end reads, the above steps must be processed for the reverse complements of input reads.

In the computational tests, *d* was changed to some extent to investigate the predictive performance of Bivartect in the benchmarking test (Supplementary Table S1). On the other hand, *d* =* *30 was used in the test with the mouse genome-editing data.

### 2.4 Removing breakpoint clusters with low quality

Closer inspection of breakpoint clusters is performed to produce high-quality predictions. First, careful attention should be paid to the ratio of predictions (having variants or not) to the total read in each sequence group of a breakpoint cluster. A normal group may contain contamination from mutated sequences, but we expect that this is very low (≤5%) in the real setting (Moncunill *et al.*, 2014). As for a mutated group, sequences from a heterozygous (heterozygous means having two different alleles for a specific trait) sample ideally include 50% of variants in the mutated group where a normal parent and a mutated parent were mated. Let $q_{\min}$ and $q_{\max}$ denote lower and upper bounds of ratios, respectively, of predicted variants to the total number of reads in the mutated group of a breakpoint cluster. For a pure condition in our benchmark data, $q_{\min}=0.9$ and $q_{\max}=1.0$ were used. In the test with the mouse genome-editing data, which contain sequences of heterozygous mice, $q_{\min}=0.35$ and $q_{\max}=0.6$ were used.

Considering the number of reads that support breakpoints also gives us an indicator for selecting high-quality variants. Let $c_{\min}$ and $c_{\max}$ be cutoffs for the minimum and the maximum number of reads, respectively, of the normal group and the mutated counterpart in arbitrary breakpoint cluster. For example, if the minimum number of reads of two groups in a breakpoint cluster is less than $c_{\min}$, the cluster is discarded in terms of few supporting reads. We fixed $c_{\min}=6$ in all computational experiments presented in this work because preliminary tests suggested that use of this value resulted in better predictive performance than $c_{\min}<6$. In contrast, $c_{\max}$ was changed to some extent in the benchmarking test (as shown by *c* in Supplementary Table S1). In the test with the mouse genome-editing data, $c_{\max}=28$ was used. Guidance on how to set Bivartect’s parameters can be found in Supplementary Notes.

### 2.5 Inferring variant type

To infer the variant type of a breakpoint cluster, the following steps are performed. Step 1: clusters that indicate the identical breakpoint are combined into one cluster by checking the identity of substrings on the left of the breakpoint. Step 2: Consensus sequences are computed in normal/mutated groups in a breakpoint cluster. Step 3: The variant type of a breakpoint is inferred by comparing normal and mutated consensus sequences. Step 4: If input reads are non-strand-specific paired-end reads, clusters that indicate the identical breakpoint on the reverse complementary strand are merged into one cluster in the forward direction. Step 5: If a breakpoint cluster of unassigned type is combined in Step 4, its variant type is inferred again. In what follows, Steps 2–5 are described in more details.

#### 2.5.1 Computing consensus sequences

In each sequence group, a consensus sequence can be computed by majority voting, resulting in a pair of consensus normal and mutated sequences (Supplementary Fig. S1). If a base to be extended cannot be determined by this approach during computation, the extension is halted.

#### 2.5.2 Inferring variant type from consensus sequences

A predicted breakpoint is checked to determine whether its type is indel, SNV, inversion or complex in that order by comparing consensus normal and mutated sequences. Examples of the complex variant type include contiguous SNVs, and a combination of SNVs and indels. These are registered as individual variants again if the complex is successfully decomposed into respective elements. If a variant is not assigned to any variant type stated above, it is judged as an unassigned breakpoint.

#### 2.5.3 Dealing with non-strand-specific paired-end reads

If there are two different breakpoint clusters that show the same breakpoint due to reads in different strand orientation (i.e. forward and reverse) across paired FASTQ files, they should be integrated into one cluster. To this end, for each breakpoint cluster, substring *s* of fixed length (e.g. 15 bp) including the breakpoint in a consensus normal sequence is cut out and stored in set *S*. Its reverse complement $\bar{s}$ is then stored in set $\bar{S}$. Similarly, sets S′ and $\bar{S\prime }$ are constructed for consensus mutated sequence s′ and its reverse complement $\bar{s}\prime$, respectively. If there are substrings t,u∈S and t′,u′∈S′ such that $t=\bar{u}$ and $t\prime =\bar{u}\prime$ where $\bar{u}\in \bar{S}$ and $\bar{u\prime }\in \bar{S\prime }$, the cluster that derives t′ and u′ is integrated into the other cluster that generates *t* and *u* (Supplementary Fig. S2).

#### 2.5.4 Re-inferring variant type for unassigned breakpoints

If two different breakpoint clusters with an unassigned variant type are integrated as described earlier, consensus sequences in the integrated cluster are likely to be extended on the basis of their pairing information, leading to the possibility of inferring the variant type of large size. More precisely, two consensus normal/mutated sequences in the integrated cluster are merged into a new longer consensus normal/mutated sequence. The two resulting normal and mutated sequences are then used to infer the variant type.

### 2.6 Post-mapping normal reads

If one needs to calculate genomic locations of predicted breakpoints, a consensus normal sequence for each breakpoint cluster is mapped onto a reference genome. To compute the positions of variants predicted by Bivartect in all computational tests, BWA-backtrack 0.7.17 (Li and Durbin, 2009) was used.

### 2.7 Constructing simulated benchmark data

First, normal paired-end reads were generated by ART 2.5.8 (Huang *et al.*, 2012) using human GRCh38 chromosome 22 as a reference sequence, where HiSeq 2500, 50, 200 and 10 were used for the built-in profile, the read length, the mean and the standard deviation of the fragment size, respectively.

Next, real mutations were downsampled from chromosome 22 of all common human variations compiled in dbSNP build 151 (Sherry *et al.*, 2001). The downsampling was done in the following order: (i) keep all SVs of length ≥50 bp; (ii) sample 200 000 variants; (iii) choose variants of the distance to their neighboring variants between 25 and 50 bp (given two variants of positions *x*_1_ and *x*_2_$\left(x_{1}<x_{2}\right)$, the distance between the two is defined as *d* such that $x_{2}=x_{1}+d$). These processes resulted in 48 281 variants in total, and the detailed numbers of SNV, indel and SV are shown in Supplementary Table S2. After integrating these variants into the reference, mutated reads were also generated by ART.

## 3 Results

### 3.1 Benchmarking Bivartect

**Fig. 2.**
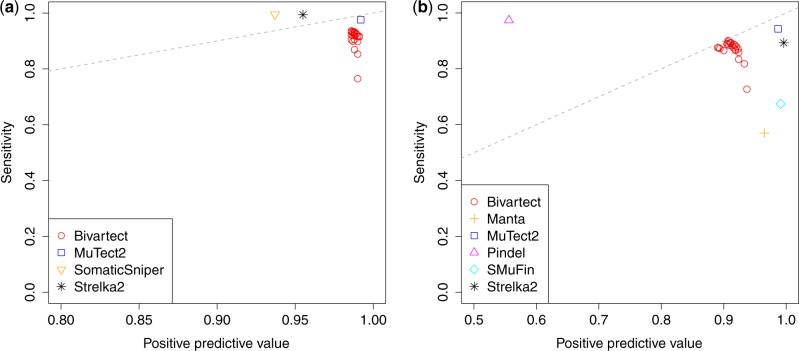
Predictive performance of each variant caller on our simulated benchmark data. (**a**) Prediction accuracy of SNV callings. (**b**) Prediction accuracy of indel callings. The 24 red points plotted in these figures correspond to Bivartect results obtained from varying the hyper parameters shown in Supplementary Table S1. Note that the dashed gray line plotted in each figure shows a *y* =* x* line. (Color version of this figure is available at *Bioinformatics* online.)

In our benchmarking test with the simulated data described above, Bivartect was compared with Manta 1.6.0 (Chen *et al.*, 2016), MuTect2 (Cibulskis *et al.*, 2013) built-in GATK 4.1.0.0, Pindel 0.2.5b9 (Ye *et al.*, 2009), SMuFin 0.9.3 (Moncunill *et al.*, 2014), SomaticSniper 1.0.5.0 (Larson *et al.*, 2012) and Strelka 2.9.10 (Kim *et al.*, 2018) as competitive variant callers. Predictive performance was evaluated by calculating sensitivity, positive predictive value (PPV) and *F*-measure, defined in Supplementary Notes. The results indicated that Bivartect achieved the high range of PPV (0.986) for SNV detection, and the third best balanced accuracy (*F*-measure, 0.904) for indels after MuTect2 and Strelka2 (Fig. 2a and b and Supplementary Table S3). Although the predictive performance of Bivartect was comparable to that of two mapping-based variant callers, Bivartect outperformed the mapping-free method SMuFin on these benchmark data.

**Fig. 3.**
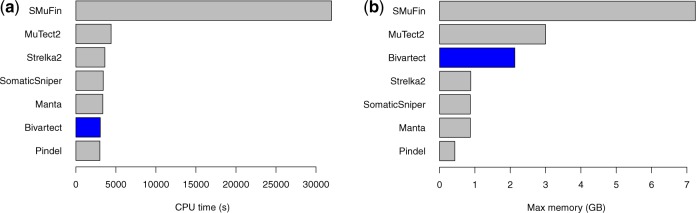
Computational performance of each variant caller. (**a**) CPU time of each tool for the benchmark data on a machine with Intel Xeon Gold 6126 (Skylake/2.6 GHz 12 cores) and 192 GB RAM. Note that all but SMuFin were run with two threads, whereas SMuFin was run with two MPI processes. (**b**) The maximum memory usage of each tool for the benchmark data. Of note, Bivartect would not be suitable for whole-genome data of higher organisms since its run-time and memory increase with data size more than those of the other mapping-based callers. See Supplementary Notes S3.3 and S3.4 for further details

We also assessed CPU time and peak memory for running the aforementioned tools by using the same benchmark data. It should be noted that time for pre-/post-mapping with BWA-backtrack was included in the CPU time. Bivartect was as fast as the other mapping-based variant callers, and 10.4-fold faster and used 3.4 times less memory than SMuFin (Fig. 3a and b and Supplementary Table S4). In addition, the peak memory use of Bivartect was 1.4 times less than that of MuTect2.

**Fig. 4.**
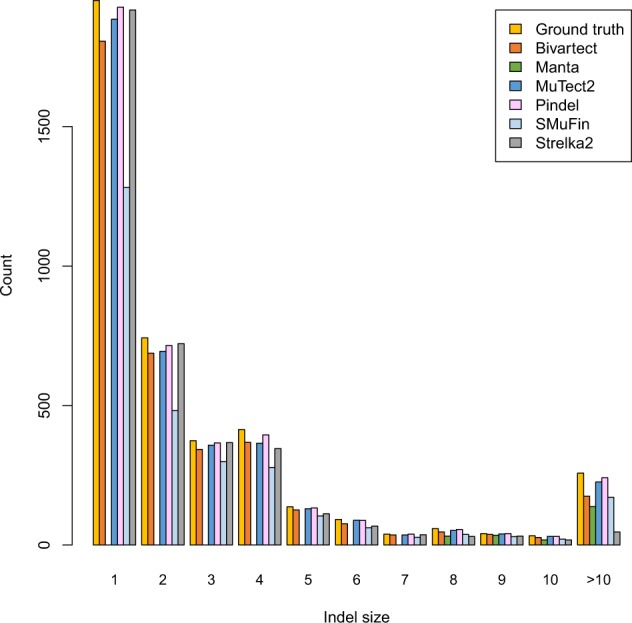
Sensitivity of each variant caller as a function of indel size on the benchmark data of 50 bp paired-end reads of coverage 30×. Note that Manta detected indels of size larger than 7 bp

Figure 4 and Supplementary Table S5 provide insight into the prediction accuracy as a function of indel size, indicating that Pindel achieved better sensitivity than the others over all indel sizes. This analysis also shows that Bivartect was better in sensitivity than SMuFin for smaller indels, and comparable for larger indels.

Next, we examined how the predictive performance of each variant caller differs with the fold-coverage of simulated reads. In this analysis, Bivartect and MuTect2 achieved the highest and the most robust PPV regardless of coverage changes for SNV detection (Supplementary Fig. S3a). For indel detection, Bivartect had a slightly lower PPV than some of the other callers, but it was still high (>0.90; Supplementary Fig. S3b). PPV of Pindel, which showed the best sensitivity on the benchmark data, tended to deteriorate as the fold-coverage increased, indicating the tradeoff between sensitivity and PPV. In addition, Bivartect is included in the leading group with respect to sensitivity for both SNV and indel detection when the fold-coverage exceeded 30× (Supplementary Fig. S3c and d). Taken together, these results indicate that Bivartect, MuTect2, SomaticSniper and Strelka2 yielded robust and accurate predictions for the coverage change.

We also investigated an effect of repeats in the genome on prediction by each variant caller. Focusing only on repeat-annotated variants in the evaluation on the benchmark data, repeats did not affect the sensitivities of Bivartect, MuTect2, Pindel, SomaticSniper and Strelka2 (Supplementary Table S6).

**Fig. 5.**
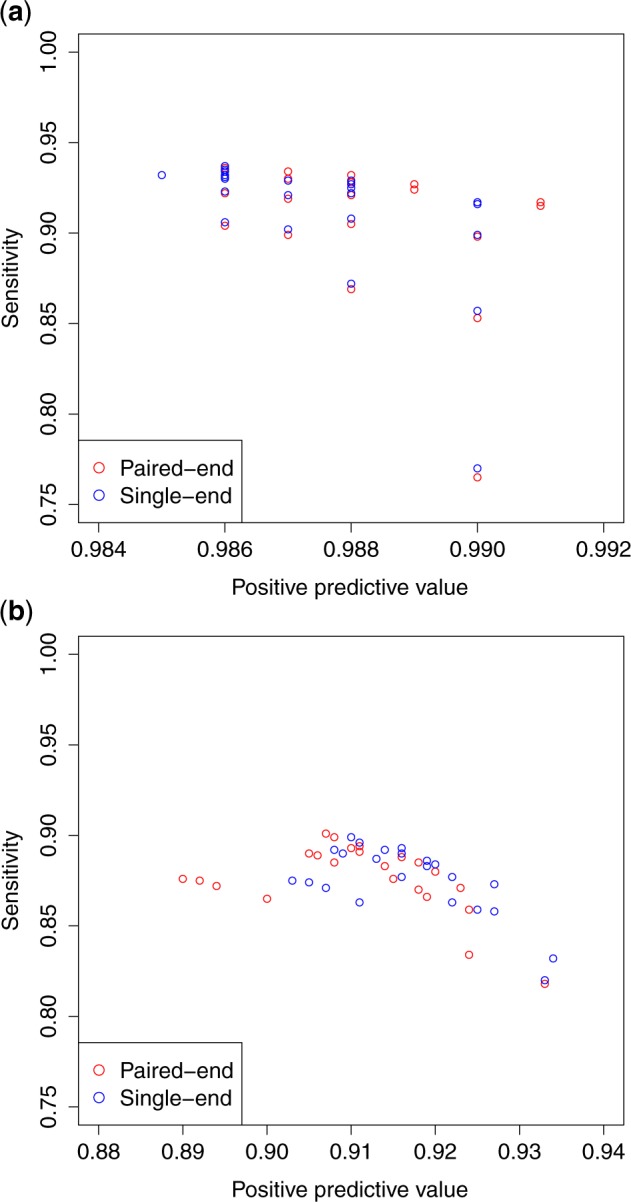
Comparison of predictive performance of Bivartect on single- and paired-end reads. Both reads are of coverage 30× and of length 50 bp. (**a**) The performance for SNVs. (**b**) The performance for indels

Unlike some other variant callers, Bivartect can also accept single-end reads as input due to its simple framework (Fig. 1). More precisely, the predictive performance for both SNVs and indels on single-end reads was comparable to that on paired-end reads (Fig. 5a and b), which is one of the advantages over some competitive variant callers that cannot deal with single-end reads.

### 3.2 Variant calling on mouse genome-editing data

We next examined the predictive performance of Bivartect relative to some leading mapping-based callers (MuTect2 and Strelka2) using real genome-editing data (DRA007211) compiled in the literature (Nakajima *et al.*, 2016). In this reported study, whole exomes of heterozygous knock-in (KI) mice carrying a single missense mutation in the *Ntrk1* gene, which were generated using CRISPR/Cas9, were sequenced with 101 bp paired-end reads of a depth 100–120× and compared with a wild-type (WT) mouse from the same breeding colony. Note that BWA-MEM was used to initially map reads when MuTect2 and Strelka2 were run. When the sequencing data of two heterozygous KI mice (Nos. 306-1 and 316-2) were compared with the WT, all three callers correctly detected four validated SNVs including an edited mutation in the *Ntrk1* gene (Table 1). However, it should be noted that the number of SNVs predicted by Bivartect was two orders of magnitude lower than predicted by MuTect2 and Strelka2. As for indels, both Bivartect and MuTect2 correctly detected one indel validated in No. 316-2, whereas Bivartect again predicted 100-fold fewer variants than MuTect2 (Table 1). Of these three tools, Strelka2 reported the fewest indels, although the validated indel was not included in the results (Table 1). These data suggest that Bivartect can detect the true variants by lining up fewer candidates, which would be an advantage in subsequent narrowing down of the potential off-target sites inserted during genome editing (Anderson *et al.*, 2018).

**Table 1.** The number of variants predicted by three tools on the mouse genome-editing data where whole exomes of two F1 heterozygous KI mice (Nos. 306-1 and 316-2) were compared with that of a WT mouse

Mouse sample (F1)	306-1				316-2			
Variant type	SNV		Indel		SNV		Indel	
	Predicted	Validated (4)	Predicted	Validated (N/A)	Predicted	Validated (5)	Predicted	Validated (1)
Bivartect	416	4	115	N/A	425	5	119	1
MuTect2	50 694	4	12 526	N/A	45 055	5	12 982	1
Strelka2	62 065	4	31	N/A	51 898	5	39	0

*Note*: The total number of validated variants is indicated in parentheses.

N/A, not available.

## 4 Discussion

Among state-of-the-art variant callers compared in this work, SMuFin, based on direct comparison of sequence reads, requires huge memory. In contrast, Bivartect not only used less memory but also achieved higher accuracy than SMuFin as demonstrated in the benchmarking test. This may be due to the simple framework of Bivartect, which is contrast to SMuFin’s complicated implementation with a message-passing interface dependent on hardware, leading to the possibility of the reduced accuracy of variant calling on the benchmark data.

The initial mapping-based approaches have a fair number of controllable parameters of constituent tools in the analysis pipeline, particularly for complex filtering after the initial mapping, whereas the direct read comparison has fewer parameters than the former due to the simple framework. In real applications, optimizing a complex set of parameters depending on the input would be hard and painstaking, and an arbitrary parameter setting (e.g. the default setting) might cause some problems such as having lower sensitivity or higher false-positive rate in the predictions. In fact, the number of predicted variants in the mapping-based callers was much larger than in the direct read comparison (Table 1). Note that Bivartect’s high PPV on the benchmark data does not necessarily mean the low number of false positives on other data. Although the number of variants detected by Bivartect in this table might be still so many that one could not validate these experimentally, information on known variants available in the future would be useful for reducing the candidates further, enabling the tool to overcome this problem. Hence, the framework of the direct read comparison would be more general and accessible than the initial mapping-based methods.

Bivartect is a memory-efficient variant caller with high accuracy, particularly for detecting SNVs, which is achieved without initial mapping. Despite its simplicity, Bivartect can handle a wide range of applications irrespective of biological species, from the detection of germline mutations in the same individual, to the confirmation of off-target mutations in genome-editing data using any exome sequence data. In addition, Bivartect is applicable to whole-genome sequence data from lower organisms, where there may not be sufficient information available on the reference genome. Details of dealing with whole-genome sequence data for higher organisms such as human are discussed in Supplementary Notes. Considering that genome-editing technologies have been rapidly utilized for different organisms, detection of variants without initial mapping is expected to increase in importance.

## Supplementary Material

btaa059_Supplementary_Data
