# Supplementary material for: Bivartect: accurate and memory-saving breakpoint detection by direct read comparison
Source: Bioinformatics. 2020 Jan 27;36(9):2725–30. doi: 10.1093/bioinformatics/btaa059 (PMC7203739; doi:10.1093/bioinformatics/btaa059)
Supplement: btaa059_Supplementary_Data [file btaa059_supplementary_data.pdf]

Supplementary Material on “Bivartect: accurate and memory-saving breakpoint detection by direct read comparison”

Keisuke Shimmura[1], Yuki Kato[1,*] and Yukio Kawahara[1]

1 Department of RNA Biology and Neuroscience, Graduate School of Medicine, Osaka University, Japan

*Correspondence should be addressed to Y. Kato (ykato@rna.med.osaka-u.ac.jp)

Contents

1 Supplementary Figures **3**

2 Supplementary Tables **6**

3 Supplementary Notes **12**

3.1 Tips on how to set Bivartect's hyper parameters 12
3.2 Evaluation measures 12
3.3 Estimating the memory requirement for a human whole genome 12
3.4 Evaluating time complexity 13

List of Supplementary Figures

S1 Computation of consensus sequences in normal/mutated groups 3
S2 Integration of two breakpoint clusters with reverse complementarity 4
S3 Predictive performance of each variant caller as a function of fold coverage on the benchmark data 5

List of Supplementary Tables

S1 The range of hyper parameters of Bivartect used for the benchmark test 6
S2 The number of variants used in the simulated benchmark data 7
S3 Predictive performance of all tools on the benchmark data 8
S4 Computational performance of all tools on the benchmark data 9
S5 Indel counts of the reference and predictions for each indel size on the benchmark data 10
S6 Sensitivity test for repeat-annotated variants 11

1 Supplementary Figures

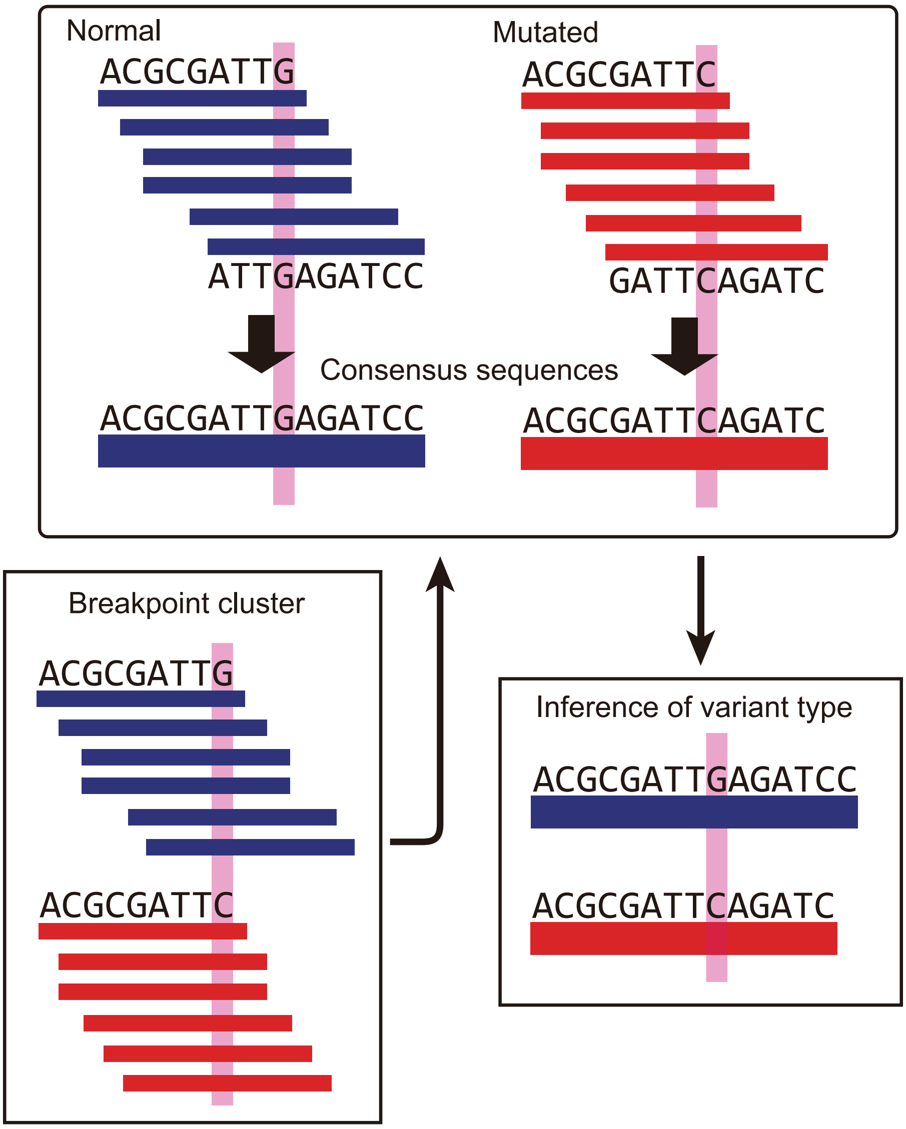

Fig. S1 Computation of consensus sequences in normal/mutated groups in a breakpoint cluster.

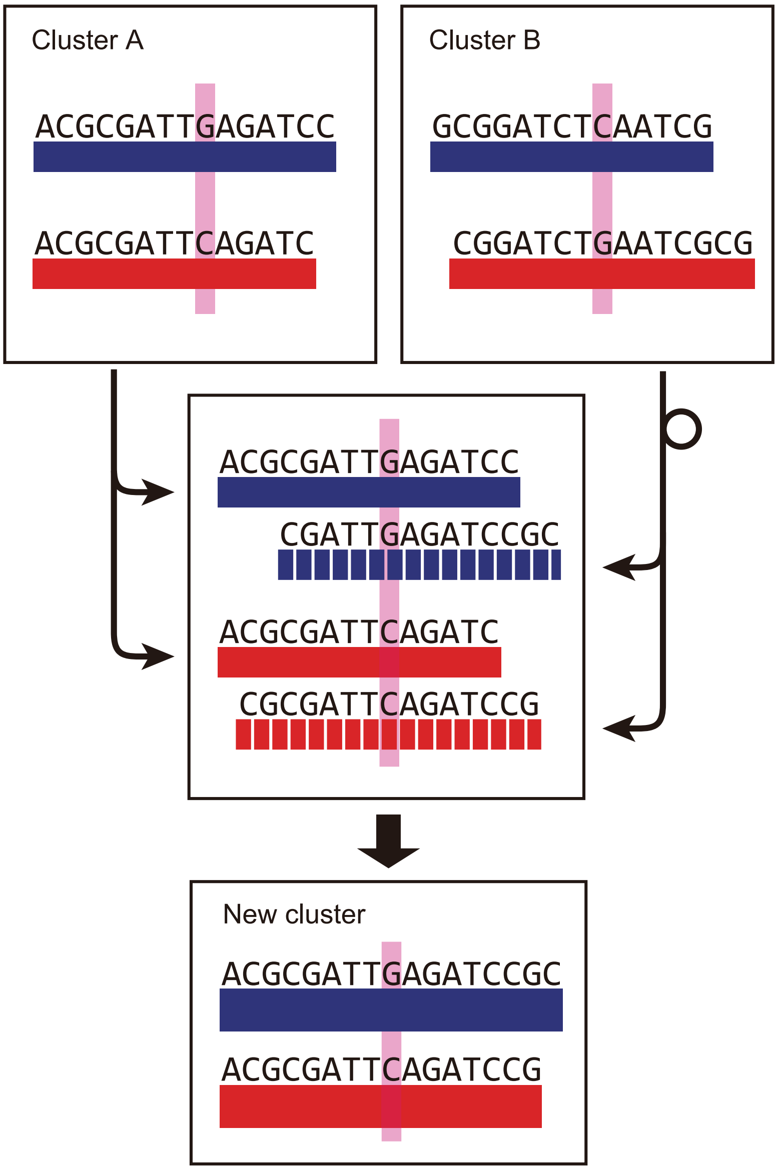

Fig. S2 Integration of two breakpoint clusters with reverse complementarity into one new cluster. Note that sequences shown here are consensus normal/mutated sequences in breakpoint clusters.

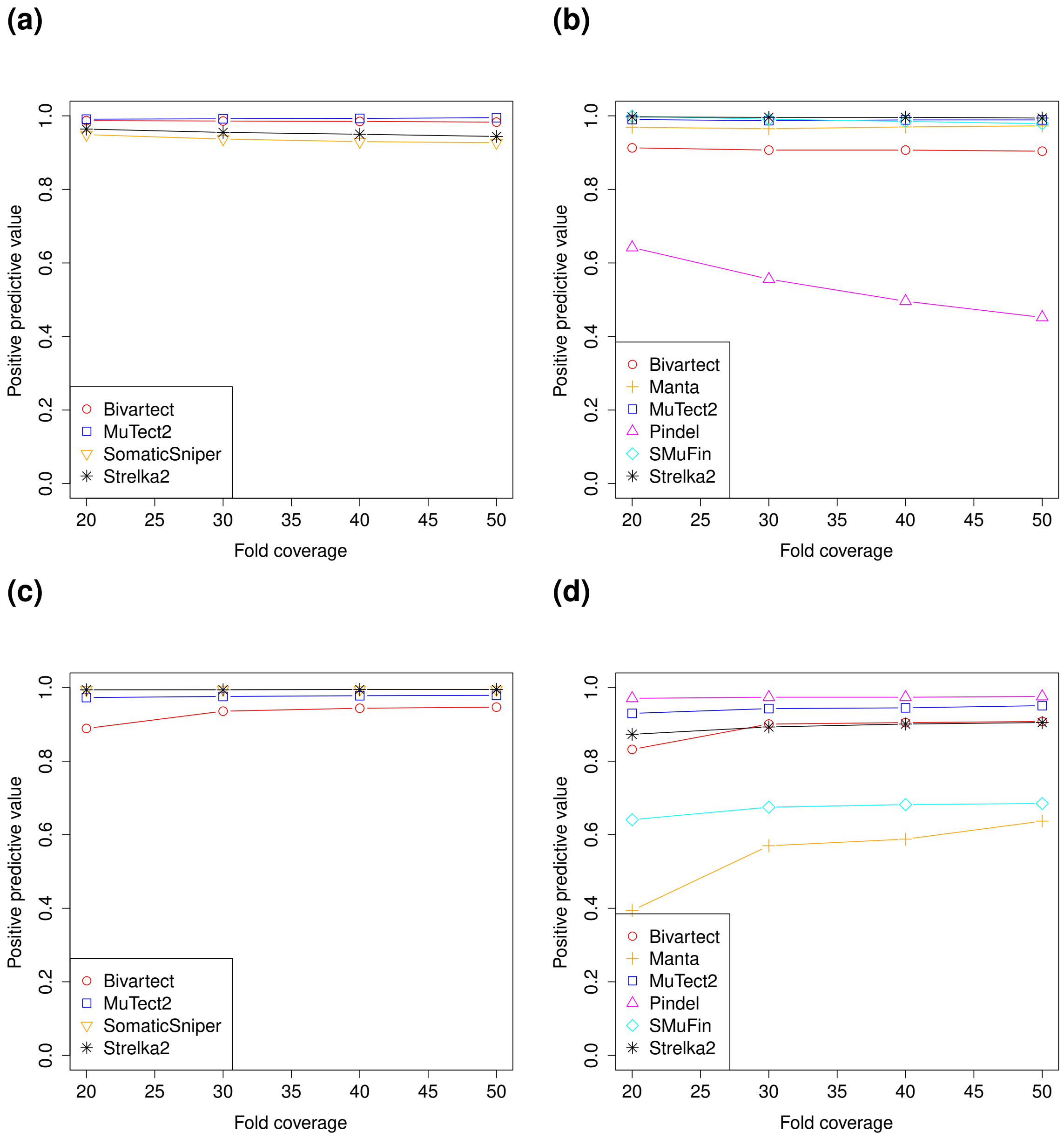

Fig. S3 Predictive performance of each variant caller as a function of fold coverage on the benchmark data of 50 bp paired-end reads. **(a)** Positive predictive value (PPV) for single nucleotide variant (SNV) detection. **(b)** PPV for small indel detection. **(c)** Sensitivity for SNV detection. **(d)** Sensitivity for small indel detection.

2 Supplementary Tables

Table S1 The range of hyper parameters of Bivartect used for the benchmark test.

Parameter	Range
Read count cutoff c	$c \in \{6, 8, 10, 12\}$
Filtering depth d	$d \in \{20, 22, 24, 26, 28, 30\}$

Table S2 The number of variants used in the simulated benchmark data. These variants were downsampled from chromosome 22 of all common human variations compiled in dbSNP build 151 [4], which were mapped to assembly GRCh38. Downsampling was done in the following order: (1) keep all structural variants of length $\geq$ 50 bp; (2) sample 200,000 variants; (3) choose variants of the distance to their neighboring variants between 25 bp and 50 bp. SNV: single nucleotide variant. Indel: insertion/deletion. SV: structural variant.

SNV	Indel	SV	Total
44,139	4,140	2	48,281

Table S3 Predictive performance of all tools on the benchmark data of 50 bp paired-end reads and of coverage $30\times$. Sensitivity, positive predictive value and F-measure are defined in Supplementary Notes. Note that Bivartect's result corresponds to one of the 24 points plotted in Figure 2 in the main article, which yielded the best F-measure with hyper parameters $c = 6$ and $d = 24$. Other hyper parameters of $p = 0.9$ and $q = 1.0$ were fixed throughout this benchmark test. The other tools used default parameters. All alignment-based methods used BWA-backtrack [3] to perform initial mapping to the reference human chromosome 22 in GRCh38. Note that predictive performance of SV is integrated into that of Indel. SNV: single nucleotide variant. Indel: insertion/deletion. SV: structural variant. SEN: sensitivity. PPV: positive predictive value. F: F-measure. N/A: not available.

Variant type		SNV			Indel	
Evaluation measure	SEN	PPV	F	SEN	PPV	F
Bivartect	0.936	0.986	0.960	0.901	0.907	0.904
Manta	N/A	N/A	N/A	0.570	0.965	0.717
MuTect2	0.976	0.992	0.984	0.943	0.987	0.965
Pindel	N/A	N/A	N/A	0.974	0.556	0.708
SMuFin	0.017	0.825	0.033	0.675	0.991	0.803
SomaticSniper	0.995	0.937	0.965	N/A	N/A	N/A
Strelka2	0.994	0.955	0.975	0.893	0.996	0.942

Table S4 Computational performance of all tools on the benchmark data of 50 bp paired-end reads and of coverage $30\times$. Note that Bivartect's results are the ones with the hyper parameters described in Supplementary Table S3.

Performance	CPU time (s)	Max memory (GB)
Bivartect	3058	2.13
Manta	3371	0.87
MuTect2	4406	3.00
Pindel	3008	0.43
SMuFin	31950	7.24
SomaticSniper	3424	0.87
Strelka2	3621	0.88

Table S5 Indel counts of the reference and predictions for each indel size on the benchmark data of 50 bp paired-end reads and of coverage $30\times$. Note that Bivartect's predictions are the ones with the hyper parameters described in Supplementary Table S3. N/A: not available.

Indel size	Ground truth	Bivartect	Manta	MuTect2	Pindel	SMuFin	Strelka2
1	1952	1806	N/A	1885	1928	1282	1918
2	743	688	N/A	694	715	482	722
3	374	343	N/A	358	366	299	367
4	414	368	N/A	365	395	278	346
5	137	126	N/A	130	133	104	112
6	92	76	N/A	89	88	62	68
7	39	36	N/A	36	39	28	37
8	59	47	32	52	56	38	31
9	41	38	35	40	41	30	32
10	33	27	18	31	31	21	18
11	24	21	16	24	24	19	13
12	22	17	15	19	21	14	7
13	25	22	20	23	24	17	9
14	18	14	12	16	18	16	5
15	14	12	8	14	14	10	4
16	16	12	7	14	15	12	3
17	13	10	6	12	12	12	2
18	15	11	6	13	15	11	1
19	8	4	4	7	8	5	0
20	11	5	4	9	11	8	0
21	13	10	4	13	13	9	0
22	11	6	7	9	10	7	0
23	6	3	2	6	6	5	0
24	11	6	3	10	10	2	1
25	8	3	4	7	8	6	0
26	5	3	2	2	4	2	0
27	6	3	2	6	6	3	1
28	1	0	0	1	1	0	0
29	2	0	0	1	1	1	1
30	4	2	4	4	4	3	0
31	3	1	2	3	2	2	0
32	3	1	0	2	2	1	0
34	2	2	2	2	0	0	0
35	1	1	1	1	1	1	0
36	1	0	0	1	1	0	0
37	1	1	0	1	0	0	0
38	1	1	0	0	1	0	0
39	3	1	1	1	2	1	0
41	1	0	0	0	0	0	0
43	1	0	0	0	0	0	0
44	1	0	0	0	1	0	0
46	1	1	1	1	1	1	0
47	1	1	1	1	1	1	0
48	2	0	2	2	2	1	0
49	1	0	0	0	0	0	0
50	2	1	2	1	2	1	0

Table S6 Sensitivity test for repeat-annotated variants in the benchmark data of 50 bp paired-end reads and of coverage $30\times$. Of note, 24,934 variants out of 48,281 ones (i.e. 52% of the original) were evaluated since their genomic counterparts were annotated with repeats by RepeatMasker. Also note that Bivartect's results are the ones with the hyper parameters described in Supplementary Table S3. Each value in the table indicates sensitivity. SNV: single nucleotide variant. Indel: insertion/deletion. N/A: not available.

Variant type	SNV	Indel
Bivartect	0.903	0.874
Manta	N/A	0.052
MuTect2	0.981	0.943
Pindel	N/A	0.973
SMuFin	0.016	0.579
SomaticSniper	0.996	N/A
Strelka2	0.996	0.879

3 Supplementary Notes

3.1 Tips on how to set Bivartect's hyper parameters

The value of $k \in \mathbb{N}$ affects run-time and memory for running Bivartect. Larger k produces more subprocesses for building a breakpoint cluster, resulting in longer run-time and less memory as compared with smaller k. For standard use, $k = 3$ is recommended because of balanced time and memory.

Optimizing $d \in \mathbb{N}$ is hard because it is associated with the trade-off between sensitivity and positive predictive value. However, the range of d shown in Table S1 would give a good candidate.

Setting $q_{\min}, q_{\max} \in \mathbb{R}$ such that $0 \leq q_{\min}, q_{\max} \leq 1$ depends on whether data are homozygous or heterozygous. If one deals with a homozygous sample, these parameters should take high values such as $q_{\min} = 0.9$ and $q_{\max} = 1.0$. In case of a heterogeneous sample, these parameters should be set around 0.5, e.g. $q_{\min} = 0.4$ and $q_{\max} = 0.6$.

The value of $c_{\min} \in \mathbb{N}$ can be fixed at $c_{\min} = 6$ as described in the main text. In contrast, $c_{\max} \in \mathbb{N}$ should be selected according to the coverage of input reads. The deeper the read coverage is, the larger value $c_{\max}$ should take. Note that the range of $c = c_{\max}$ shown in Table S1 would be appropriate for a coverage between 20$\times$ and 50$\times$, while a large value such as 28 would be good for a coverage of 100$\times$.

3.2 Evaluation measures

Predictive performance on the benchmark data was evaluated by calculating sensitivity (SEN), positive predictive value (PPV) and F-measure (F), defined by

$$\text{SEN} = \frac{\text{TP}}{\text{TP} + \text{FN}}, \quad \text{PPV} = \frac{\text{TP}}{\text{TP} + \text{FP}}, \quad \text{F} = \frac{2 \times \text{SEN} \times \text{PPV}}{\text{SEN} + \text{PPV}},$$

where TP is the number of true positive variants, FP is the number of false positive variants, and FN is the number of false negative variants. By definition, the range of these measures is from 0 (worst) to 1.0 (best).

3.3 Estimating the memory requirement for a human whole genome

The theoretical space complexity of the Bivartect's algorithm is evaluated as $O(nl)$, where n is the total number of normal and mutated reads, and l is the maximum length of the reads. In fact, for the benchmarking 50 bp paired-end reads of coverage 30$\times$ from human chromosome 22,

$$\begin{aligned} \text{estimated maximum memory} =& (11744261 \times 2 + 11743987 \times 2) \times 50 \\ =& 2.3 \text{ [GB]}, \end{aligned}$$

where 11,744,261 and 11,743,987 are actual numbers of normal and mutated reads in one of the two paired-end read files, respectively. This estimation gives a good upper bound of the memory usage as compared with the Bivartect's result (2.1 GB) shown in Supplementary Table S4.

We are now ready to focus on the memory usage on a human whole genome. Let us take a whole genome sample NA12878 in the 1000 Genomes Project Data [1] as a simple example, which was also used in MuTect's paper [2]. Of note, the downloadable sequence read data were actually downsampled from the originals of coverage 30$\times$, and the data that we use here contain 92,459,457

reads of length 101 bp and of coverage 5× in one of the two paired-end read files. Assuming that the number of control reads is the same as that of NA12878 reads, Bivartect will require

$$\begin{aligned}\text{estimated maximum memory} =& 92459457 \times 4 \times 101 \\ =& 37.4 \text{ [GB]}\end{aligned}$$

for the human whole genome data of coverage 5×. Considering that the standard coverage for whole genome analysis will be at least 30×, this estimation tells us that Bivartect can run on a machine with at least $37.4 \times 6 = 224$ [GB] memory for a common configuration of human whole genome analysis.

3.4 Evaluating time complexity

The theoretical run-time of Bivartect is evaluated as $O\left(nl \log(nl)\right)$, because the dominant part in computation is to sort $O(nl)$ combined normal and mutated read suffixes, and $n \gg l$ can be assumed in general. In contrast, a read alignment algorithm that a mapping-based approach uses can run in $O(nl)$ time. That is why Bivartrect would be infeasible to run on a human whole genome of large n due to the additional multiplicative $\log(nl)$ term as compared with initial mapping-based methods.

References

[1] The 1000 Genomes Project Consortium (2015) A global reference for human genetic variation. *Nature,* **526**, 68–74.

[2] Cibulskis,K., Lawrence,M.S., Carter,S.L., Sivachenko,A., Jaffe,D., Sougnez,C., Gabriel,S., Meyerson,M., Lander,E.S. and Getz,G. (2013) Sensitive detection of somatic point mutations in impure and heterogeneous cancer samples. *Nat. Biotechnol.,* **31**, 213–219.

[3] Li,H. and Durbin,R. (2009) Fast and accurate short read alignment with Burrows–Wheeler transform. *Bioinformatics,* **25**, 1754–1760.

[4] Sherry,S.T., Ward,M.H., Kholodov,M., Baker,J., Phan,L., Smigielski,E.M. and Sirotkin,K. (2001) dbSNP: the NCBI database of genetic variation. *Nucleic Acids Res.,* **29**, 308–311.
